# The Automation Technique Lab-In-Syringe: A Practical Guide

**DOI:** 10.3390/molecules25071612

**Published:** 2020-04-01

**Authors:** Burkhard Horstkotte, Petr Solich

**Affiliations:** Department of Analytical Chemistry, Charles University, Faculty of Pharmacy, Akademika Heyrovského 1203, 500 05 Hradec Králové, Czech Republic; Petr.Solich@faf.cuni.cz

**Keywords:** Lab-In-Syringe, automation of sample pretreatment, potentials and troubles, system setup and operation modes, tips and tricks in method development, 3D printing of instrument elements

## Abstract

About eight years ago, a new automation approach and flow technique called “Lab-In-Syringe” was proposed. It was derived from previous flow techniques, all based on handling reagent and sample solutions in a flow manifold. To date Lab-In-Syringe has evidently gained the interest of researchers in many countries, with new modifications, operation modes, and technical improvements still popping up. It has proven to be a versatile tool for the automation of sample preparation, particularly, liquid-phase microextraction approaches. This article aims to assist newcomers to this technique in system planning and setup by overviewing the different options for configurations, limitations, and feasible operations. This includes syringe orientation, in-syringe stirring modes, in-syringe detection, additional inlets, and addable features. The authors give also a chronological overview of technical milestones and a critical explanation on the potentials and shortcomings of this technique, calculations of characteristics, and tips and tricks on method development. Moreover, a comprehensive overview of the different operation modes of Lab-In-Syringe automated sample pretreatment is given focusing on the technical aspects and challenges of the related operations. We further deal with possibilities on how to fabricate required or useful system components, in particular by 3D printing technology, with over 20 different elements exemplarily shown. Finally, a short discussion on shortcomings and required improvements is given.

Academic Editor: Pawel Koscielniak

## 1. Introduction

The term “Lab-In-Syringe” ambiguously describes two analytical techniques that have been developed within the last decade and given the same name. Both take advantage of a syringe to carry out sample preparation procedures. In the first approach, one or two, generally disposable, syringes are used to facilitate manual procedures while the second one refers to an automation technique, i.e., employing an automatic computer-controlled syringe pump. In both cases, the benefit is taken from the fundamental characteristics of a syringe: (i) size-adaptability, (ii) a steadily sealed compartment, (iii) ability to measure and transfer fluids, (iv) and the ease of emptying and wiping action of the piston on the barrel walls. In both cases, the syringe voids are used as mixing, reaction, and extraction chambers, most often to carry out liquid-phase microextraction (LPME) procedures.

In this article, we deal exclusively with the automation technique “Lab-In-Syringe” and present a tutorial on the system setup, operation modes, and required materials as well … with the as a critical overview of its applicability, potential, and shortcomings. 

Lab-In-Syringe (LIS) derives from the far better-known flow technique Sequential Injection Analysis (SIA) described already in 1990 by Růžička and Marshall [1,2]. Flow techniques (FTs) are versatile automation tools for laboratory procedures consisting of, e.g., solution metering, mixing, dilution, and analyte conversion into detectable forms. As the name suggests, these unit operations are carried out in flow within a tubing manifold and modulated by means of pumps and valves and generally by the aid of a liquid carrier that transports the injected sample and reagent solutions. 

Usually, flow systems integrate appropriate flow-through detectors (optical, electrochemical, …), achieving specific advantages in terms of analysis speed, reproducibility, and reliability as well as for analyte discrimination, e.g., via chemometrics. These are enabled by precise reaction timing, reproducible detector feed, controlled detector passage, and precise timing, so that, e.g., contact times of the analyte with immobilized enzymes or electrode surfaces are strictly reproducible. Alternatively, FT can be coupled to external instrumentation, e.g., HPLC or atomic spectrometric techniques, and used for preceding automated sample preparation by matrix elimination and analyte preconcentration. Determination selectivity of FT is achieved by tactical employment of selective reagents and enzymes, by kinetic differentiation, or by the hyphenated selective detection or separation techniques. For the latter two, FT can contribute significant advantages as a versatile tool for sample processing, by carrying out required analyte conversion, derivatization, or enrichment. 

Typical chemical problems solved by FT are the automation of analytical assays applied to all kinds of liquid samples such as waters, beverages, body fluids, or fuels. Most often, FT are applied to inorganic analytes, such as nutrients, metal contaminants, or total indices (total phenols, MBAS, DIC, …). For organic compounds, analyte selective enzymes (glucose—glucose oxidase), selective chromogenic reactions (formaldehyde with acetylacetone), or simple matrix elimination (gas diffusion for volatiles) are explored as well as taking advantage of more or less unique analyte’s reactivity (e.g., ascorbic acid) or physical properties (e.g., a fluorescence activity). FT are ideal for the automation of reactions of fast kinetics, e.g., chemiluminescence assays, and versatile tools for monitoring of a sample stream originating from technical, environmental, or biological processes. One major trend is the automation of SPE or LLE procedures for sample cleanup and analyte enrichment for online-coupled instrumental techniques ICP, GFAAS, or HPLC, among others. 

FT automation generally yields a significant gain in repeatability, sample throughput, and assay reliability and allows miniaturization compared to manual processing and reduction of sample and reagent volumes. Processing in a closed tubing system also implies a low risk of user’s exposure to harmful reagents or sample contamination, as well as automatic system cleaning by the carrier flow. 

The various FT and proposed methodologies differ in manifold configurations, operation characteristics and flow patterns, arrangement and types of valves and pumps, flow segmentation, modes of sample insertion, and obtained transient signals [3,4]. It is impossible here to give a picture of the many facets of FT with justified thoroughness. Therefore, earlier treatises and tutorials on the matter are recommended [5,6,7,8]. To introduce to Lab-In-Syringe, it is, however, necessary to elucidate at first two earlier FTs: SIA and the Flow-Batch concept. 

SIA is based on a simple computer-controlled system consisting of a single bidirectional pump, typically an automatic syringe pump, and a selection valve with all required solutions and the detector connected on its lateral ports. A long tube of a dead volume that often exceeds the volume of the employed syringe, the so-denoted holding coil, connects the common port of the multiposition valve to the syringe pump. It serves to intermediately contain the subsequently aspirated sample and reagents upon which the solution zones penetrate and mix partly. The stacked solutions undergo further mixing and reaction during flow reversal and on their way towards the detector yielding a tailing peak. This way, chromogenic reactions can easily be automated and miniaturized. Procedural parameters (volumes, flow rates, timing, pausing) are easily adapted, enabling high operational flexibility. Of special interest can be steps with stopped-flow for prolonging reaction times or monitoring only the progress of a chromogenic reaction or formation of concentration gradients that are enabled by the incomplete mixing of the stacked solution zones undergoing further dispersion in-flow. On the other hand, homogenous mixing of widely different volumes of solutions, reactions requiring multiple reagents and steps, or maintaining mixing patterns in case of varying sample viscosity are less feasible to accomplish. Here, air-bubble segmentation of a confined zone of stacked solutions can be used to achieve homogenous mixing of small solution volumes. 

Another option is the return to what has been denoted “beaker-chemistry” [3], i.e., the placement of a mixing chamber, such as the barrel of a disposable plastic syringe, on one lateral port of the selection valve. This enables the stepwise addition and mixing of solutions as needed, in other words, “batch-processing” but operated via the sequential injection analyzer, leading to “flow-batch analysis” [9]. The chamber, particularly if constantly agitated, enables homogenous mixing, widely independently from the initial sample viscosity, or open port sampling, e.g., using a pipette to introduce the sample to the flow analyzer. However, in the lack of a carrier flow, there is also a higher risk of carry-over effects and, using an atmospherically open mixing chamber, of contamination. In consequence, tedious cleaning is required often, if not even after each analysis. Chambers and the flow-batch concept have been implemented not only in SIA but also in combination with other FT, and for other purposes. A comprehensive review on this topic is recommended [10]. 

Flow-batch is one step towards discrete analyzers, i.e., liquid handling on versatile autosamplers or, more and more, by robotic arms that allows mimicking “hu-man-ual” handling of samples [3,11]. For the automation of very fast reactions, e.g., using chemiluminescence as detection principle, the study of reaction kinetics, membrane separations, online digestions, or continuous monitoring 24/7 and fieldwork, FT are ideally suited tools and superior to batch automation. However, a critical comparison of both automation concepts is apposite considering sample preparation. In batch and flow-batch automation, the preparative procedure, e.g., an LLE approach, can essentially be broken down into steps of stepwise addition of solutions, homogenous mixing, and liquid withdraw. Consequently, the workflow and procedural parameters are far more predictable than if mixing is based on dispersion-based flow patterns. It seems fair and reasonable to aim for combining both flow and batch-processing if automation of sample pretreatment procedures is intended, would it not be for the addressed shortcomings of flow-batch analysis.

Removing the holding coil from an SIA system violates a fundamental principle of this FT as the solutions will enter the syringe void from where they cannot be flushed out completely as they undergo turbulent mixing. Either severe carry-over or repeated syringe cleaning must be therefore accepted. However, intending to use the syringe as a controlled mixing chamber, flow-batch analysis is feasible in a simplified and more compact SIA system and, as it will be discussed, with increased efficiency while omitting several of its inherent weaknesses. Through the marriage of flow-batch and SIA concept, a new and versatile automation approach was created that enables new operation modes and suits ideally for accomplishing liquid-phase microextraction approaches and that has been now widely recognized by the term “Lab-In-Syringe” [12,13]. 

## 2. Lab-In-Syringe—Technical Milestones

To the best of our knowledge, it was Maya et al. who “misused” in 2012 for the first time the void of an automatic syringe pump to automate an analytical procedure, in concrete, dispersive liquid-liquid microextraction (DLLME) of benzo(α)pyrene into octanol [12]. In contrary to manually performed DLLME, the procedures started by aspiration of a mixture of water-immiscible extraction solvent and miscible dispersion solvent into the syringe followed by a very fast aspiration of the aqueous sample. The turbulence led to solvent dispersion and formation of small droplets observed as the characteristic cloudy state. After phase separation by droplet floatation and spontaneous coalescence, the analyte-enriched organic phase was subjected to low-pressure chromatography. 

In the following, steps of analyte derivatization and complexation were added to determine copper and aluminum in water samples as hexanol-soluble complexes with bathocuproine and lumogallion, respectively [14,15]. As in flow-batch, a mixing chamber was added to the flow manifold to enable homogeneous mixing of the sample with the required reagents before carrying out in-syringe DLLME that consequently required intermediate cleaning. 

**Figure 1:**
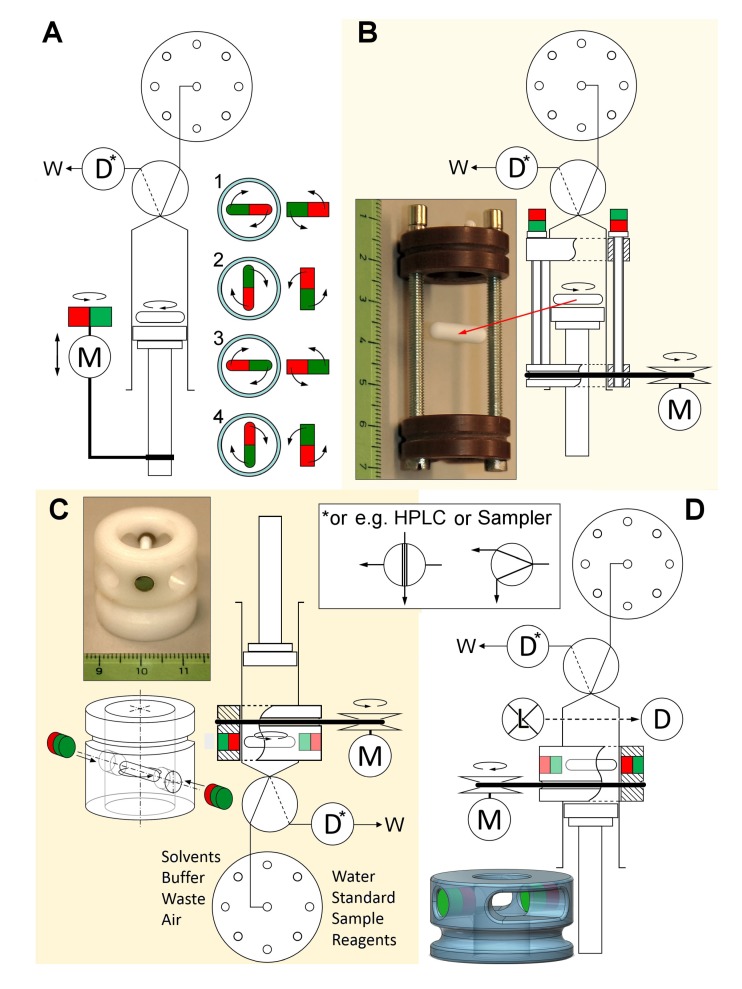
Modalities for system setup, syringe orientation, and stirring arrangements. Stir-drivers are shown in photographs with a levitated magnetic stirring bar. (**A**,**B**,**D**): Syringe in upright orientation, (**C**) Syringe used upside down. (**A**) Stirring induced by a closely placed motor with an attached bar magnet, (**B**) Stir-driver for the full stroke length of the syringe, (**C**,**D**) Stir-driver for fixed position. Upon turning the stir-drivers via a rubber ring and motor, they generate a rotating magnetic field around the syringe barrel. Abbreviations D—Detector, L—Light source, M—Motor, W—Waste.

Therefore, an important step in LIS development was the option of in-syringe homogenous mixing by using a magnetic stirring bar inside the syringe void. Possible modes of how to induce stirring are summarized in Figure 1. 

In the first work reporting this approach, the stirring bar was forced to spin by a stir-driver as shown in Figure 1B [16]. It integrated two oppositely magnetized iron rods that were placed onto the syringe barrel to create an external rotating magnetic field along the stroke-length of the syringe on demand by the aid of a relay-controlled motor. The field was strong enough for rotation speeds of 1000 rpm. The superiority of this approach over using a mixing chamber was proven by reoptimizing the earlier extraction of aluminum-lumogallion but omitting the dispersion solvent and disrupting the extractant into fine droplets by the kinetic energy of the stirring bar instead. In effect, a lower solvent consumption and detection limit were achieved in a shorter time [15,16] and instantaneous homogenous mixing for differently viscose samples was verified. Hereafter, the suitability of LIS for (i) online standard preparation by adaptable in-syringe stock dilution and (ii) automation of a procedure involving 12-steps and 10 solutions for the derivatization and DLLME of chromate as Cr(III)-diphenyl carbazone complex was shown [17].

The resulting inability to empty the syringe completely was solved by simply turning the syringe upside-down. By this, air remained inside the syringe above the liquid level and expelled all solution content from the syringe during emptying (Figure 1C). The cost of this advantage is that each liquid transfer is delayed by the compressible air cushion that necessarily remains inside the syringe. Nonetheless, this arrangement enabled the reproducible performance of automated DLLME procedures into solvents denser than water while previous works had all used floating solvents. The classical assay of total cationic surfactants, requiring chloroform, was miniaturized and automated this way [18]. Noteworthy is that it was possible to use a much simpler stir-driver since the magnetic bar was not displaced with the syringe piston but remained at the bottom, near the syringe inlet. This enabled a focused magnetic field and higher rotation speeds of up to 3000 rpm that exceeded the typical stirring velocities in manual stirring-assisted DLLME severalfold. 

Using the syringe itself as a detection cell by the aid of a fiber optic adaptor (see Figure 1D and Figure 4) was yet another milestone set by Maya et al. in 2012 [13] that justified the denomination “Lab-In-Syringe”. This potentially increases the system simplicity and compactness, but a limitation is given by the requirement of using visible and near-UV wavelengths which can pass through a glass syringe. 

Another lift in technical versatility was featuring a secondary entrance to the syringe void by drilling of a longitudinal channel through the syringe piston that enabled accessing the headspace created inside the syringe [19,20] or using the syringe as flow-through reactor [21]. 

Finally, it was by Anthemidis’ working group to incorporate for the first time a secondary syringe pump into a LIS system and to use syringe heaters to enhance both liquid-gas transfer and reaction kinetics in the determination of ammonia with *ortho*-phthaldialdehyde [22].

## 3. Lab-In-Syringe—Characteristics, Potentials, and Limitations

In terms of technical versatility as automation- and flow technique, the following features and advantages of using a syringe as mixing, reaction, and extraction chamber are noteworthy:

(1) The syringe in a LIS system is a permanently sealed compartment, thus user exposure to reagents or sample contaminations during processing are avoided similarly as by using a tubing manifold.
(2) The syringe represents a size-adaptable void allowing both transferring and containing liquids, gases, and even sorbent suspension that makes it an ideal tool for the automation of laboratory procedures that require pipettes, burettes, and flasks. Consequently, the syringe now executes these functions for acting both as the pump and the mixing chamber in a very compact system.
(3) Size adaptation and the wiping action of the syringe piston on the inner wall of the syringe barrel also implies that the small remaining space of the emptied syringe requires cleaning. Therefore, this step can be done more efficiently and faster than in SIA-operated flow-batch either by fast aspiration of the cleaning solution with mixing by resulting turbulence or by in-syringe magnetic stirring. Using SIA for flow-batch operation, the entire chamber must be filled with the cleaning solution. Moreover, it should be stressed out that each solution transfer requires, in fact, two steps, e.g., one for sample aspiration into the holding coil and a second for sample transfer into the mixing chamber.
(4) The size adaptability allows changing of the pressure inside the syringe if the syringe’s head valve is turned to a permanently closed position. This can promote analyte evaporation at reduced pressure [19,22,23,24] or to solvate a gaseous compound at increased pressure [22].
(5) Placing a magnetic stirring bar inside the syringe allows using a far higher stirring rate than in an open chamber where the liquid content would be lost at vigorous stirring by splashing. Moreover, in-syringe stirring can be performed at any stage of the procedure and prolonged as needed. Also, presence of air bubbles or immiscible phases, i.e., extraction solvents, varying sample viscosity, or mixing of very small with very large solution volumes are without difficulty while in tubing-based FT, each item alone can present a significant challenge. Finally, in-syringe stirring allows for efficient void cleaning between analyses. Summing up, we consider it a waste of potential if choosing LIS for procedural automation but without in-syringe stirring. In this context, we draw attention to Figure 1 summarizing the modes to induce in-syringe stirring.
(6) The usual glass syringes are transparent, allowing its use as a cuvette for photometric detections. Even if detection is done outside the syringe, mixing the solutions homogeneously means that the Schlieren effect can be avoided, which is often troublesome in dispersion-based FT [25].
(7) Finally, a drilled-through piston enables (i) a second entrance to the syringe void [19], (ii), linkage to hyphenated instrumentation [21], and (iii) using the syringe as a flow-through reactor [21].

Summing up, LIS allows, in contrast to tube-based FT, reproducible treatment of samples of several milliliters in a sealed, size-adaptable reactor that enables the addition of further reagents, on-demand homogenous mixing, gas-liquid separation, in-situ detection, pressure adaption, and is more efficient in terms of system cleaning compared to the flow-batch approaches. Despite this versatility, there are still certain disadvantages against FT based on tubing manifolds:

(1) The main disadvantage is the large dead volume of the syringe that requires cleaning after each analysis. This step can take > 1 min or up to 30% of the total procedural time while most FT are “self-cleaning” by the action of a carrier flow. Performing this step efficiently is discussed in the previous listing, item 3 and section 5.12.
(2) Reproducible formation of a concentration gradient “in-space” is impossible while it is easily formed in the holding coil of an SIA system just by aspiration of different solutions. This tool can be used to determine two analytes of distinct reaction kinetics depending on the chemical milieu or to compensate for matrix effects. On the other hand, there is the possibility to aspirate solutions either stepwise or gradually into the syringe so that a gradient “in-time” is still feasible.
(3) Solution cooling and heating are more efficient in a thin tubing manifold than as for the bulk solution inside the syringe yet syringe heating to promote analyte volatilization has been reported recently using a resistance wire wrapped around the syringe barrel [22]. Earlier, we integrated an efficient heater into the short holding coil to enhance reaction kinetics [14].
(4) Similarly, sample digestion has been, to our knowledge, not yet accomplished inside the void of a LIS system. Classical pretreatment techniques based on membranes, i.e., gas-diffusion, are equally not feasible in-syringe yet the suitability for automation of head-space extraction can partly compensate for that.
(5) The main advantages of the flow-batch techniques, i.e., FT based on using an open mixing chamber, over LIS is that a sensing element, e.g., an electrode, can easily be integrated into the system (placement into the chamber), that open-port sampling is enabled, i.e., interfacing the analyzer with manual pipetting or other instrumentation, and that reactions that generate gases cannot induce critical overpressure.

## 4. Modes of Operation and Automated Methodologies

In the following, we give an overview of the possible operation modes and methodologies that have been automated using Lab-In-Syringe focusing rather on technical challenges than aiming for a comprehensive review of previous applications. Coupling LIS to other analytical instrumentation will be also addressed. 

In the first place, the simplicity of automation of standard laboratory procedures, i.e., chromogenic assays, by LIS should be pointed out. In contrast to most FTs, mixing a large sample volume with step-by-step added reagents is straightforward and does not require such optimization as studying solution stacking and zone dispersion. Mere downscaling of an existing protocol and using the pre-optimized reagents is likely to work from the start. As the only complication, in-syringe solution heating could be quoted but was reported howbeit [14,22]. Since nearly instantaneous homogenous mixing can be achieved using a stir-driver, measurement before reaching the reaction steady-state can be considered possible, similarly as in another FT. 

Only a few papers report on the automation of only chromogenic assays, all abstaining from in-syringe stirring but using an additional mixing chamber, stepwise aspiration of solutions, or air for solution homogenization inside the syringe. Using an almost identical system as in early LIS works, Ma’s group reported on the analysis of ammonium and chromate in waters [26,27]. While the LIS concept was applied and cited, the authors invented their own flowery expression of “integrated syringe-pump-based environmental-water analyzer (iSEA)”. In two works from Koscielniak’s group, in-syringe mixing was integrated into FT analyzers for flexible preparation of sample solutions for iron determination studying chemometric tools for reduction of matrix effects [28,29]. Finally, the LIS-automated determination of ester content in biodiesel was reported where ethanol was used for in-syringe preparation of a homogenous phase of the hydrophobic sample and aqueous reagents to accomplish a chromogenic reaction [30]. 

**Figure 2:**
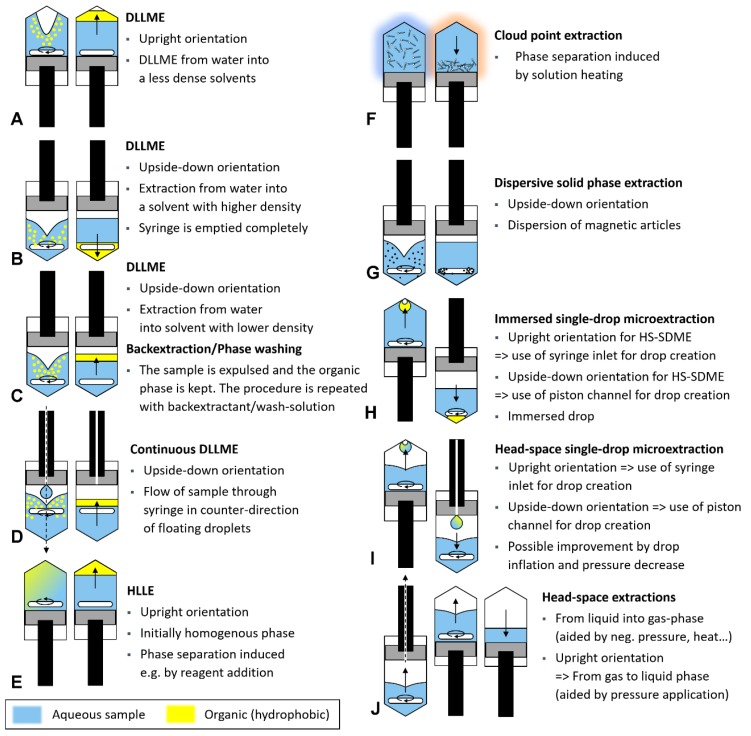
Overview of sample pretreatment procedures automated by Lab-In-Syringe with in-syringe stirring. In **A**–**G**, the left scheme indicates the extraction phase while the right one shows phase separation, in **H**–**J**, different modes of a similar methodology or operation are shown. Black arrows indicate the direction of analyte transfer, dotted back arrow indicate flow.

Most of the developed analytical applications based on LIS have focused on the automation of various sample pretreatment procedures, which are summarized schematically in Figure 2. We also refer to commented videos on Youtube channel “In-Syringe Analysis/Lab-In-Syringe”. 

It is out of scope to represent and discuss all operation modes in a tutorial so that we chose those which added technical or procedural novelty no aiming for a comprehensive listing. Three review articles on automated liquid phase sample preparation are recommended instead [31,32,33].

Among all LIS applications, automation of LPME approaches and, in particular, DLLME, has been reported most often. Solvents both lighter and denser than water have been used following the schemes indicated in Figure 2A,B, respectively. Ideally, the phase to be used for detection or injection into an online coupled instrumentation is expulsed first to avoid carry-over so that an upright syringe orientation is advantageous for using a floating solvent and vice versa. Such systems have been used repeatedly ones combining LIS-DLLME either with mixing chambers or in-syringe stirring. Spectrophotometric detection has been used mostly taking advantage of analyte-selective chromogenic reactions or ion-pair formation [17,18] including utilization of liquid waveguide capillary cells [14], in-syringe detection [13], or fluorescence measurement [15,16]. 

Moreover, the online coupling of LIS-DLLME of phthalates and UV-filters to GC-MS via a micro-injection valve was reported including in-syringe silylation inside the extractant [34,35]. DLLM-extract collection for posterior scintillation counting of the enriched 99Tc was reported where LIS automation significantly improved the time efficiency of an otherwise tedious sample cleanup [36]. 

Generally, phase separation is based on droplet floatation/sedimentation and spontaneous coalescence. However, this approach works best and fastest for solvents of low viscosity and in the absence of surfactants, which can cause the formation of stable emulsions. In this sense, Maya and co-workers demonstrated an alternative approach and collected 1-dodecanol droplets by in-flow solidifying in a 3D printed Peltier cooled phase separator that was handled by simple robotics [37].

LIS-DLLME has been further coupled online to atomic spectrometric techniques ETAAS [38] and ICP-AES [39] achieving stirring-assisted solvent without any stir-driver (see Figure 1A). To achieve compatibility of extractant xylene with ICP-AES, a heated nebulization chamber had to be used [39]. An alternative to the direct injection of the organic extract and a mode that circumvents compatibility problems of the coupled instrumental technique is analyte back-extraction equally automated in-syringe. Using a floating solvent, an upside-down orientation of the syringe allows keeping the organic phase inside while discharging the sample. Hereafter, an appropriate reagent can be aspirated for dispersive back-extraction (Figure 2C) with posterior photometric detection [21,40] or coupling to ICP-AES [41]. For a solvent denser than water, the organic phase can be “parked” in the connected manifold to enable the complete discharge of the sample or, consistently, the syringe is used upright. A washing step for the elimination of remains of the sample matrix can be carried out between sample discharge and back-extractant aspiration. Here, mixing by turbulence suffices while renewed stirring and consequent solvent dispersion will prolong the procedural time by pausing for phase separation. This step can also be used for solvent washing for which a lower stirring rate can be advantageous to avoid long phase separation times [18]. 

Recently, we demonstrated DLLME with back-extraction converting the syringe into a flow-through chamber that was enabled by a channel in the syringe piston (Figure 2D). The sample was entering the syringe from above and flowing out below while a loss of solvent was hindered by keeping the velocity of the sample flow lower than the floatation speed of the solvent droplets [21]. Concerning alternatives to classical liquid-liquid extraction and related solvents, ionic liquids and deep eutectic solvents have been used in LIS-automated LPME methodologies. For instance, UV-filters were extracted into an ionic liquid and separated by coupled HPLC. An additional syringe was used in this case for extractant dilution prior to injection [42]. Elsewhere, a deep eutectic solvent was used as disperser as a modification of earlier reported chromate extraction as diphenylcarbazone-Cr(III) complex [17,43]. 

Bulatov’s group has reported on LIS-automated homogenous liquid-liquid extraction (HLLE, Figure 2E). HLLE starts with a homogenous phase of the sample and a water-miscible solvent that exhibits better compatibility with liquid chromatography than classical extraction solvents. Phase separation is induced, e.g., by the addition of salt, change of pH, or addition of a tertiary solvent [31]. An important advantage of HLLE is that emulsion formation and loss of extraction solvent on matrix components such as particles are neglectable compared to the relatively large volume of used solvent and even only moderately hydrophobic compounds can be extracted efficiently. In a first work, phase separation of acetonitrile-water was induced by the addition of a concentrated glucose solution that enabled the extraction of pesticides from juices and separation by online-coupled HPLC [44]. In the second work, a switchable solvent (DEHPA) was used that formed a homogenous phase with the sample under alkaline condition (DEHPA ionized). Phase separation was then induced by aspiration of acid into the syringe to extract antimicrobial drugs before HPLC separation [45].

Moreover, in-syringe automated cloud point extraction (CPE) has been reported twice using Triton X-114 as the surfactant (Figure 2F) for the extraction of colored analyte derivates of antimony and epinephrine with subsequent spectrophotometric detection. In the first work, required solution heating to yield phase separation and enrichment of antimony as iodide complex was achieved by in-syringe dilution of high concentrated sulfuric acid [46]. A heating block was used in another work reporting CPE of epinephrine where the syringe was used for phase separation and abscission [47].

Maya and co-workers reported on LIS-automated dispersive solid-phase microextraction on model analyte malachite green and estrogens. They used a magnetic nanostructured sorbent (metal-organic framework) inside the upside-down oriented syringe for this purpose (Figure 2G). The sorbent particles were dispersed by in-syringe stirring while stopping the stirring, the magnetic sorbent was attracted to the stirring bar. The sample was easily exchanged first by a washing agent to remove any sample remains and then by a suitable eluent, all without risking a loss of sorbent particles [48,49]. 

Two works report on LIS-automated extraction into a drop of extraction solvent that is in direct contact with the sample. Advantages are a simpler setup since fast stirring was not required and omission of emulsion formation as low stirring speed suffices and does not impair drop integrity. A drop of solvent, that simply floated on the aqueous sample and was stabilized by the stirring vortex, enabled extracting silver as thiocarbamate complex with subsequent analysis by online connected ETAAS [50]. In another work, lead was determined by spectrophotometry after being extracted as dithizone complex comparing two approaches for drop formation (Figure 2H) [51]. With the syringe upright, a drop of floating solvent clang on the syringe inlet and was stabilized by an air bubble inside. For the second approach, the syringe was used upside-down and a drop of chloroform was used with a lentil-shaped stirring cross turning slowly inside the drop. The new approach of “in-drop stirring” was superior: Both drop stabilization and surface movement that enhanced analyte transfer were achieved and a stirring time of only 150 s was sufficient for quantitative extraction.

An interesting feature of LIS is the gas-tightness and size adaptability of the syringe, which enabled the automation of various sample pretreatment methodologies involving gas-liquid transfer as well as positive and negative pressure applications. In-syringe head-space single-drop microextraction (HS-SDME, Figure 2I) was first applied to the selective determination of ethanol in wines using a mixture of sulfuric acid and chromate as chromogenic drop reagent that turned green at the reaction with the evaporated ethanol from the sample inside the syringe. The process was supported by negative pressure application and inflating the drop surface by a small bubble inside [23]. Adding a second syringe pump, the precise formation of a drop of palladium nanoparticle suspension was done by Anthemidis’ group to extract elementary, thus volatile, mercury from the sample with subsequent transfer of the enriched drop of Pd nanoparticles to ETAAS [24]. In a third work, contact of the drop with the head-valve manifold was strictly avoided. The syringe pump was placed upside-down and a drop of indicator solution was generated inside the headspace by pushing the reagent with a second pump through a drilled channel in the first syringe piston. The color change of the drop was then measured online by fiber optics allowing the determination of ammonium in surface waters [19]. 

Finally, headspace extraction, gas transfer, and capturing gaseous compounds into an appropriate reagent is feasible (Figure 2J). Online coupling of a simple LIS system to GC-FID was reported that allowed the transfer of 80% of the gaseous phase after in-syringe headspace enrichment with the volatile analytes (benzene, toluene, ethylbenzene, xylene). This simple procedure yielded similar sensitivity than former reports requiring a secondary analyte enrichment or trapping [20]. 

In a LIS system combing two syringes, ammonia was forced to volatilize in syringe 1 that was assisted by heating and negative pressure application. Subsequently, the analyte was captured and let react with the fluorogenic reagent *ortho*-phthaldialdehyde in syringe 2 that was assisted equally by heating and by compression of the transferred gas [22]. Finally, for the determination of dissolved inorganic carbon, in-syringe capturing of released CO_2_ was done by an indicator solution [52].

## 5. Tips and Tricks for System Setup, Method Development, Optimization, and Characterization

**Figure 3:**
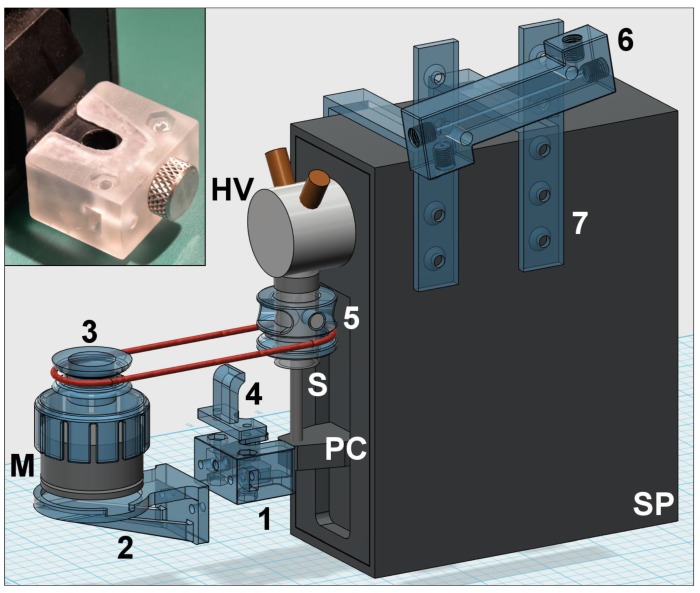
Lab-In-Syringe system from a syringe pump (SP) with 2 port head valve (HV) with an upright oriented syringe (S). 3D printed elements are shown in blue color and disassembled, required screws are not shown. A simple stir-driver (5) is connected to a motor (M), featured from a computer fan moving, with a removable top-attachment with pulley wheel (3) via a rubber band. On the piston carriage (PC), a universal adapter (1) allows fixing a holder for the motor (2) and a lifting lug (4) to move stir-driver and motor upwards with the syringe piston. An SLA-printed 5 cm detection flow cell (6) is mounted on top of the syringe pump using a universal adaptor (7). The photo shows the SLA-3D printed adaptor (item 1) for easy mounting additional elements to the piston carriage.

**Figure 4:**
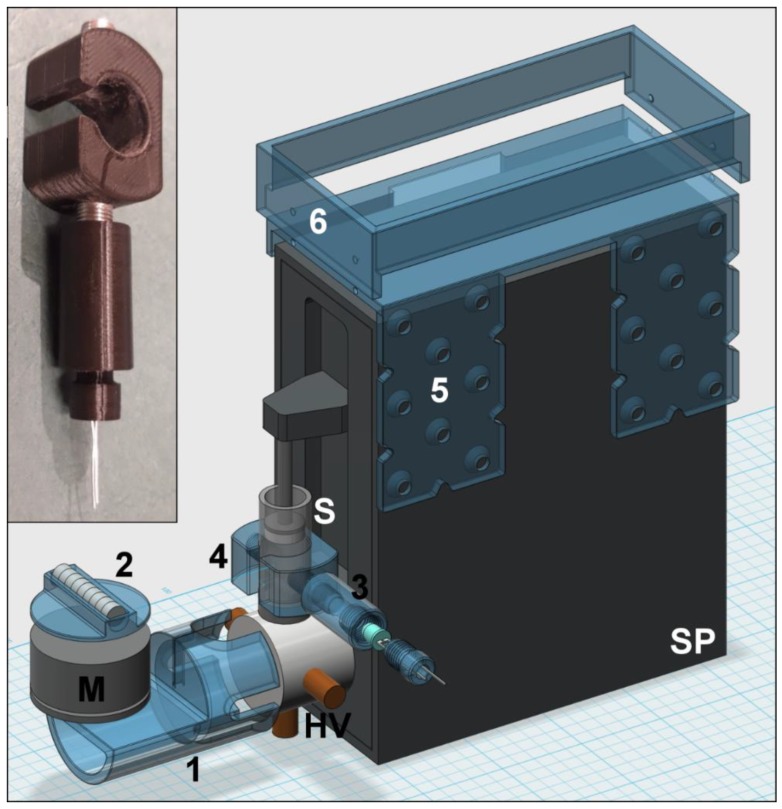
Lab-In-Syringe system from a syringe pump (SP) with 3 port head valve (HV) with an upside-down orientated syringe (S). 3D printed elements are shown in blue color and disassembled, required screws are not shown. A removable adaptor for the head valve (1) allows the positioning of the motor (M) close to the syringe. A holder for NdFeB magnets (2) is glued on-top that induce stirring bar rotation inside the syringe at the motor start. A LED holder (3) is screwed onto a fiber-optic adaptor (4) that is placed onto the glass barrel for in-syringe photometric measurements (Photometer and optical fibers not shown). Elements 5 and 6 compose a tray and universal support for deposition of tools (fittings, screwdriver, etc.) and to attach further items, e.g., the relay board for motor control. The photo shows the 3D printed elements 3 and 4 for in-syringe spectrophotometry.

**Figure 5:**
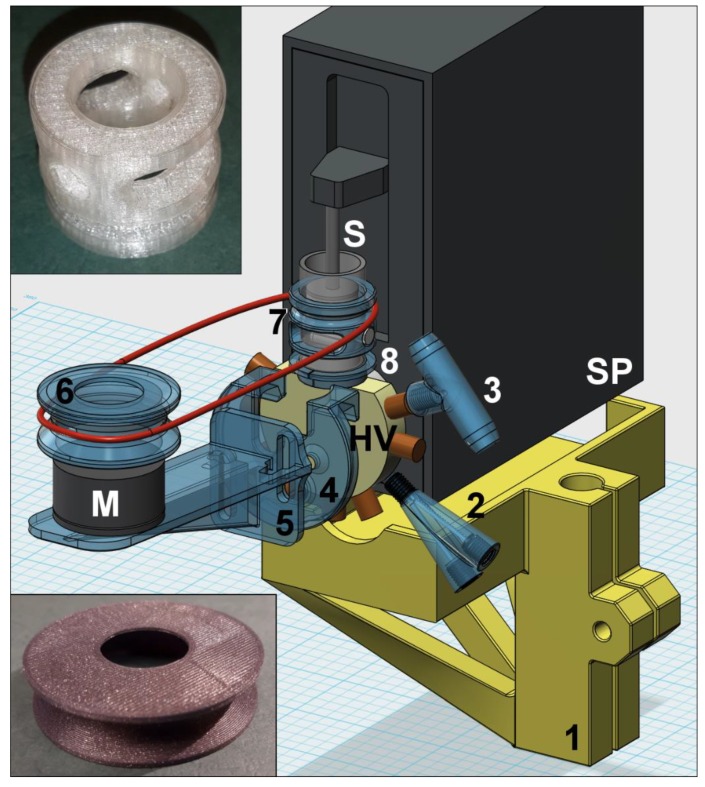
Lab-In-Syringe system from a syringe pump (SP) with nine port head valve (HV) with an upside-down orientated syringe (S). 3D printed elements are shown in blue color and disassembled, required screws are not shown. The syringe is situated on a laboratory stand compatible support (1). On the head valve, SLA-printed elements add the functionalities of a flow-through port (2) and connecting a drain tube to waste as well as air aspiration (3), respectively. Elements 4 allow mounting further elements on the head valve, here a support (5) for positioning and leveling of the stirring motor (M). The motor is equipped with a pulley-wheel (6, photography below) connecting the motor via a rubber band to a simple stir-driver (7, photograph above) that is held in place by a supporting ring (8).

After this overview that demonstrates the high versatility of Lab-In-Syringe, in this section we give tips and tricks that should assist in the instrumental setup and use of the LIS technique. Setups of Lab-In-Syringe systems including 3D printed system elements are shown in Figure 3, Figure 4 and Figure 5.

### 5.1. Syringe Orientation

One of the first steps surely is to decide on how to position the syringe. Upright syringe orientation or the use of a secondary syringe are advantageous if highly precise liquid handling or pressure application is required or for such DLLME procedures using a solvent with lower density than water (Figure 2A). For DLLME using a floating solvent, it has been also proven most helpful to aspirate air into the syringe to create an open liquid surface. This enables vortex formation during in-syringe stirring and facilitates the dispersion of the extraction solvent.

Vice-versa, upside-down syringe orientation will be more suitable for dispersing a solvent that is denser than water (ionic liquids, halogenated solvents, deep eutectic solvents, etc.) as the stirring bar will be situated on the bottom of the syringe and close or inside the solvent and promote dispersion (Figure 2B). This setup has also proven useful if a floating solvent is used for a procedure involving back-extraction so that the sample must be expulsed first (Figure 2C). 

Often, we found the upside-down syringe orientation to be the better choice as it allows emptying the syringe nearly completely and the syringe piston is not in contact with the sample. This can be advantageous in the case of particulate sample matrix or hydrophobic compounds sticking to PTFE. To minimize friction and eventually mechanical wearing of the piston in this arrangement, add a drop of glycerol as lubricant into the upward opening of the syringe barrel. 

In upside-down orientation, the user must plan that each liquid propulsion must be followed by at least 1–2 s of pausing due to the compressibility of the air cushion inside the syringe that delays any liquid movement. In consequence, this setup is less suitable for handling viscose liquids or for small volumes. Moreover, tubing of larger or equal diameters than 0.8 mm i.d. should be used in combination with a 2.5 mL syringe to reduce the formation of negative pressure at aspiration at higher speeds and solution degassing. 

Before system and method setup, preliminary experiments on the expected extraction conditions (pH, ion-pairing reagent, etc.) and, foremost, to select possible extraction solvents (density, stickiness, etc.) is surely advantageous as this will decide on syringe orientation. The optimization is then started using the same solutions and volumetric ratios as used in the standard procedure or tested.

### 5.2. Solvents and Chemicals

There are hardly any limitations on usable solvent or reagents as contact surfaces of the syringes are typically glass and inert plastics. However, the syringe piston will eventually be attacked by the solutions inside the syringe so that regular cleaning of the syringe piston is recommended. In case of high ionic strength of sample or reagents, incrustation of salt must be prevented/removed as it will damage the syringe piston with time. In principle, the same rules apply as for seal wash in liquid chromatography. Low viscosity, low water solubility, high vapor pressure, and a significant difference in density towards the aqueous sample are beneficial features of a solvent intended for DLLME procedures to achieve efficient solvent dispersion, phase separation, and droplet coalescence and to avoid degassing or solvent loss by dissolution in the aqueous phase. If stable emulsions are formed, nondispersive approaches such as DI-SDME will be more suitable. One imitation to be highlighted is that hydrophobic solvents tend to stick to the PTFE surfaces of syringe piston and inlet. Here, cleaning with moderate hydrophobic solvents such as isopropanol, diminishing the inlet diameter (see Figure S1), and an upside-down syringe orientation are helpful. 

### 5.3. Syringe Model, Holding Coil, and Head Valve

We have worked for a long time with Cavro^®^ automatic syringe pumps of 3 cm and 6 cm stroke length with rotary head valves (Tecan Trading AG, Switzerland) but there should be no limitations regarding the syringe pump model. Pressure stability > 2 bar of the syringe head valve is highly desirable to avoid problems related to limited robustness and all materials in contact with liquid should be chemically inert (PTFE, glass, PEEK, etc.). 

The holding coil is a system component that will be required only occasionally and should generally be short to maximize the volume that can actually be used for the in-syringe accomplished procedure. Complete omission of a holding coil is possible using syringe pump models that feature a multiposition head valve (six or nine ports). It is indeed astonishing how much these pumps have proven fit-for-purpose so that it remains an open question whether they were originally intended for some in-syringe automation. 

However, even on a 9-port head valve, the lateral ports are rapidly occupied for waste disposal, water, cleaning solution, sample, a detector or secondary instrument, e.g., HPLC, and possibly standard solution for in-syringe addition, buffers, solvents, etc. To build up positive or negative pressure inside the syringe, a permanently closed position is further required. Finally, aspiration of air might be needed and is highly useful to enable vortex formation inside the syringe, gas-liquid separations, or aspiration of all liquid from the holding coil into the syringe void. 

The required position for waste and air can be combined in particular if the syringe is used in an upside-down position. For this, a PEEK tube of only a few centimeters is connected to the selection valve and pierces on its other side a wide tube (several millimeters) that acts as a drain to waste. This way, the dead volume required for waste disposal is minimal and, considering that in this syringe orientation, the air is the last thing to be expulsed from the syringe at content discharge, aspiration of any undesired solution with air is neglectable. An adapter for this purpose is shown in Figure 5.

### 5.4. Syringe Dimensions

The length-to-diameter ratio of the syringe can be of some importance. For example, a slender syringe will facilitate the separation and removing the organic from the aqueous phase while exhibiting a small surface area in case of gas-liquid transfers. In the case of headspace extraction, a wider diameter at equal syringe volume will be advantageous. In respect to the syringe size, one important argument for choosing a smaller one is this allows building up a higher pressure with the available force of the syringe pump, which might be a limiting factor in some applications. Decision criteria can further include the available sample size or the aimed preconcentration factor. Finally, the shape and material at the transition zone between syringe inlet and barrel should be contemplated; a smooth outflow of both liquid and bubbles at emptying the syringe is highly desirable. Moreover, we would recommend a syringe diameter so wide that in-syringe stirring is feasible and air bubbles cannot segment the liquid column inside the syringe. 

### 5.5. Syringe Inlet

To minimize the dead volume in the syringe inlet we have proposed to insert a piece of 0.8 m tubing as shown in Figure S1B that also increases the flow velocity and turbulence for stirring-less in-syringe DLLME [14]. To increase the hydrophilicity of the syringe inlet we have used a glass capillary to stabilize a liquid drop in the syringe inlet for headspace single-drop microextraction [23].

### 5.6. Motors

Slow-turning motors can be taken from old VCR devices. For faster speeds, we have good experience featuring motors from computer fans as shown in Figure S2A–D. Models with pulse width control have proven more reliable in terms of speed stability and starting power at low speed. Moreover, they are easily regulated and brushless, i.e., they show a far longer lifetime than cheap hobby motors which will fail after a few weeks. Higher priced models are suitable for high spinning speeds and show generally a greater momentum than the ones from computer fans. Several analog control circuits have been described [16,18,41]. In the long term, power control by a microcontroller as recently proposed [37] is surely the way how to improve the reliability of motor operation, a key issue of LIS. In Figure 3, Figure 4 and Figure 5 and Figure S2E we show 3D printed motor supports as well as motor attachments that either facilitate fixation of magnets on top of the employed motor or that serve as a pulley wheel if a stir-bar driver is utilized and must be forced to rotate via a rubber band.

### 5.7. Stirring Without Stir-Driver

We can only recommend using the approach of in-syringe stirring since we consider the gain in versatility higher than its backsides, i.e., a higher dead-volume and required system extension. In all cases, we used NdFeB magnets and commercial stirring bars for this purpose with positive results. 

The simplest option to accomplish in-syringe stirring is to place a motor of low to a moderate velocity close to the syringe with a pile of disk magnets fixed on top. Adding or removing magnets then allows adapting the magnetic force (see Figure 1A and Figure 4). In consequence, the stirring bar inside the syringe will follow the rotation speed of the motor. For simple mixing at low spinning speed, there is no need for computer control so that this option is ideal for the automation of bare chromogenic assays or headspace extractions [20,23]. To avoid tumbling or bouncing of the stirring bar, a short stirring bar or a stirring cross (Figure S1D) that is a fair approximation to a round disk are ideal. The exact placement of the motor using such stirring bars will be of low importance. 

If higher speeds are desired, we found that the force of the stirring bar and of the magnetic pile must be higher and the motor must be placed closer to the syringe. However, in such a case it is crucial that the motor exhibits a high momentum at its start. If too weak, the magnetic pile and thus the motor are retained by the attraction from the stir bar inside the syringe. We recommend using a stirring bar that fits smoothly into the barrel and to start at low rotation speed before accelerating [18,41]. The motor must also be lifted with the piston to assure leveling of the magnetic pile and bar magnet and avoid stir bar bouncing. Optimization will take some effort yet stirring fast enough for DLLME are feasible. 

### 5.8. Stir-Driver

Alternatively, an element that attracts and consequently aligns the stirring bar from its both sides can be used, which we have denoted, in lack of a better term, “stir-driver”. It is forced to rotate around the syringe axis by the motor via a simple rubber ring (See Figure 1B–D, Figure 3 and Figure 5) purchasable at any stationery store. Evenness, close-to-round profile, and > 15 cm of circumference are fit. These rubber rings degrade in laboratory air and must be exchanged at times. 

Two kinds of stir-drivers have been proposed so far that can be produced with little effort in a tool shop or from hardware store material. Using the syringe in an upright position, the stirring bar must move up and down with the piston it is laying on so that the creation of a magnetic field along the full stroke length of the syringe is required. As shown in Figure 1B, only two plastic rings, one with an annual groove for the rubber band, that fits smoothly over the barrel, two long iron screws, and two smaller NdFeB magnets are needed. A thorough description can be found elsewhere [16]. 

A simpler stir-driver can be used when the stirring bar remains in the same position, typically when the syringe is used upside-down [18]. Here, a single plastic ring, e.g., featured from a piece of PVC water pipe, with two holes for the two, NdFeB magnets opposing each other and an annular groove is required as shown in Figure 1C. An improved version is easily produced by 3D printing that allows adding cut-outs for better observation of the inner part of the syringe as shown in Figure 1D. As the magnetic force is more focused in this design, stirring rates up to 3000 rpm are possible without that the stirring bar “loses track” but rotates synchronously with the driver. At such high velocities, the use of a dispersion solvent should not be required anymore. Moreover, the NdFeB magnets levitate the stirring bar inside the syringe so that friction is minimal and lifting the stir-driver will allow dislocating the stirring bar to the perfect position, e.g., the boundary between organic and aqueous phases as previously described [21]. 

### 5.9. In-Syringe Detection

The simple stir-driver or a driver-less system setup leave space for using an optical-fiber adaptor (see Figure 1D and Figure S1E) to enable in-syringe spectrophotometric detection [13,19]. The advantage is that there is no need for solution transfer that might cause baseline alterations by Schlieren formation. In any case, it is recommended to use a detection cell from material that is easily wettable by the phase of interest, i.e., glass for the aqueous, polymers for the organic one. A 3D printed element is shown in Figure 4.

### 5.10. Piston Channel

Accessing the syringe via a secondary inlet can be of use for phase separation, converting the syringe into a flow-through reactor, or creation a hanging drop inside the syringe void [13,19,20]. After drilling the piston longitudinally, a short PEEK tube can be glued in with silicone or special adhesive (Figure S1C). If not required, the piston channel can be easily closed with a short piece of silicone rubber tube as a connector and a simple plug.

### 5.11. Worn Out Piston Head

With time, the piston head of each syringe wears out by friction, abrasion by particles or salt crystals, and it will no longer seal sufficiently. As it is generally made of PE or PTFE that deform under pressure, a certain improvement can be achieved by pressing the piston head on a flat and smooth surface with a slightly circling motion that will bell out the piston head to a slightly wider diameter and achieve tighter sealing. 

### 5.12. Syringe Cleaning

This step is easily done by repeated aspiration of 20–25% of the syringe volume of an appropriate cleaning solution with activated in-syringe stirring and expulsion to waste immediately or after a few seconds. As a universal approach we recommend cleaning subsequently with a pure or diluted miscible solvent (e.g., with acid or base, possibly mixed in-syringe), and water. If the syringe is in an upright orientation, i.e., it cannot be emptied completely, the final step can be performed with the intended sample if sufficient in amount to minimize undesired sample dilution. Using the syringe upside-down, additional aspiration of air and stopping the stirring during emptying is recommended to efficiently empty the syringe.

### 5.13. Optimization

The order of aspiration and placement of solutions on the selection valve should be carefully contemplated to minimize carry-overs and turning times. The less important solution or the largest volume should be aspirated last so that any aspiration error causes a relatively small variation in the analytical result. 

During in-syringe mixing of chlorinated solvents and aqueous sample, we have observed the formation of a slight overpressure that builds up due to spontaneous degassing. In upside-down orientation and solution heating, e.g., at the addition of methanol to water, the headspace will expand and equally increase the pressure inside the syringe slightly. This can deteriorate the reproducibility of the aspirated volume in this setup. The problem can be minimized, e.g., by slow aspiration of air as the final step, applying negative pressure with the head valve turned to a permanently closed position, or saturate the solvent with water. 

Expectable dependencies of the signal intensity on selected experimental parameters for a LIS-automated DLLME procedure are shown in Figure S3 so to help newcomers in respect of what to expect during method optimization. Extraction and back-extraction times follow simple saturation behavior while a stepwise increase will be observed for the stirring speed where it becomes fast enough to achieve solvent dispersion. Time of phase separation can be visually controlled and so that generally no further optimization study is needed. While phase separation is typically achieved in 20-60 seconds depending on the solvent density, spontaneous droplet coalescence will vary with the solvent viscosity and can require additional time. It must be also taken into account that a part of the solvent will dissolve in the aqueous phase and depending on the ionic strength that will differ between standard and samples (Figure S3). To achieve high reproducibility, low solvent solubility, increasing the ionic strength by salt addition, or the use of a solvent volume that surpasses the solubility in water several times, is therefore required. 

We would refrain from optimizing all parameters by experimental design and at once. The user must also rely on his own experience and reasoning to decide which parameters’ behaviors and interactions are difficult to foresee, thus requiring such optimization, while other parameters can be fixed first to higher, “save-side” values and evaluated later, possibly in univariant studies, e.g., the stirring rate. The choice of the final conditions should not only aim for the highest performance but also the high reliability, robustness, and comparability between standard and matrix-loaded samples. 

In method development it must be considered that any magnetic stirrer inside the syringe will decrease the applicable stroke length by a few millimeters, i.e., the usable void volume will decrease by a few hundred microliters. If using the syringe in upside-down orientation, this volume is then occupied by the air remaining in the syringe. 

### 5.14. Head-Space Extractions

In the automation of HS-SDME, it should be kept in mind that even using the syringe upside-down, cleaning the syringe by stirring will also wet the piston head and can imply undesired contamination of the channel inlet, requiring e.g., a slower stirring rate. On the other hand, creating a drop in the normal entrance of the syringe [19,20,21] implies an even larger risk of drop contamination. The use of a secondary syringe pump of much smaller dimensions for precise drop formation has been, therefore, a significant advantage [21,24].

### 5.15. Determination of Performance

Preconcentration factors and extraction efficiency can be calculated from Equations (1), (2), and (3). We would like to highlight that the effective preconcentration cannot be calculated from the analytical signals but rather the analyte concentrations found in the sample and the extract. For their calculation utilizing the analytical signal, the detection sensitivity for the analyte in both sample and extraction medium must be taken into account.

Potential preconcentration factor = V_Extract_/V_Sample_ (1)

Effective preconcentration factor = c_Extract_/c_Sample_ = Signal_Extract_ × Sensitivity_Sample_/ (2)

Signal_Sample_ × Sensitivity_Extract_

Extraction efficiency = c_Extract_ × V_Extract_/c_Sample_ × V_Sample_ (3)

### 5.16. Determination of Stirring Rate

We recommend using a laser tachometer that can be purchased for less than 30 Euro. If a stir-driver is used, the reflective foil is fixed on the stir-driver or the ratio of the diameters of the pulley wheel and the stir-bar driver must be taken into account. For low stirring rates, the use of a bicycle tachometer is possible as it uses a hall sensor so that measurement is based on moving magnets. However, the high rotation speeds often used for in-syringe stirring are likely to be outside the typical application range of such devices so that the results might be unreliable.

### 5.17. Determination of Dead Volumes

For characterization of a system as given in Figure 1B (syringe is upright) it can be useful knowing the dead volumes of the holding coil and the space inside the syringe that cannot be emptied due to the stirring bar. For this, we recommend the following:

(1) The absorbance of a solution of a highly water-soluble, stable, and colored substance (e.g., 10 mmol/L potassium dichromate) is measured in a dilution of 1:10 offline in a glass cuvette, yielding Abs1. If the LIS integrates spectrophotometric detection, the syringe is cleaned several times with the solution that is then finally passed through the flow cell.
(2) A 1:10 mixture of dye solution and water is then prepared in-syringe. The syringe and holding coil must be cleaned and initially filled with water and the syringe piston at its highest position. First, the dye solution is aspirated from one lateral port of the selection valve and then the volume of water from a second port. After mixing, the syringe content is pushed out through an empty channel, collected and measured likewise, yielding Abs2. The volume of water that was not aspirated into the syringe corresponds to the volume that has been originally inside the holding coil so that an additional dilution is caused only by the water that was already inside the syringe (the dead volume to be evaluated).
(3) The procedure is then repeated but starting with an empty holding coil, yielding Abs3. If correctly performed, the absorbance values should decrease in the order Abs3 > Abs1 > Abs2. From these values, the dead volumes can be calculated by Equations (4), (5), and (6) abstaining here from any detailed mathematical derivation:

Vs_yringe_ = (Abs1−Abs2)/Abs2 × Dilution ×·V_Dye_ here, Dilution = 10 (4)

V_total_ = V_Water_ · 1/(1/(Abs3/Abs1 × V_Dye_/(V_Dye_ + V_Water_) – 1)) (5)

V_holding coil_ = V_total_ − Vs_yringe_ (6)

## 6. Contributions to Lab-In-Syringe by 3D Printing

Lab-In-Syringe is perhaps the latest offspring of FT and finding commercial support is thus difficult. On the other hand, there are certain elements required for system setup, first and foremost the stir-driver, that appear to require a specialized tool shop. However, fabrication of the required elements that include supports for motor and syringe pump, stir-drivers, motor attachments, e.g., a pulley-wheel for forcing the stir-driver to rotate via a rubber band or a magnet holder, as well as elements in contact with solutions can easily be accomplished by 3D printing. 

The two most commonly used and economic 3D printing techniques that fully comply with all needs are stereolithography (SLA) and fused deposition modeling (FDM). Printers based on both techniques are available for about 300 Euro. Now, there are so many users that the likelihood is high that a printer can be found in the circle of acquaintances. Besides, the printing material is cheap and adequate designing software for element design can be downloaded for free or used online. 

In SLA, the object is created layer-by-layer from a monomer solution that polymerizes/hardens where it is illuminated by a laser or an LED matrix. The technique yields high surface quality, high special resolution, and liquid-tight sealed structures, making SLA useful for elements in liquid contact. However, the choice of available polymers is limited, solvent resistivity is rather low, and only smaller objects can typically be printed. 

FDM deposits molten material, provided as a filament, in thin strings that are laid out so close to each other that a solid object is created, row-by-row and layer-by-layer. The selection possibilities of materials and printable sizes are far larger but obtaining liquid-tight layer-bonds and smooth surfaces require some optimization or post-print treatment, e.g., solvent vapor smoothing. The technique is ideal for printing supports, motor attachment, and stir-drivers. In fact, we found that SLA printed stir-drivers had a velvety surface that exhibited too much friction for proper operation. On the other hand, FDM prints with PLA showed the required slippery, smooth, and hard surface. 

In Figure 3, Figure 4 and Figure 5, we show and explain three exemplary LIS setups with about 20 elements that were 3D printed using FDM technology (if not indicated otherwise) as well as successfully tested and used for LIS applications. Selected 3D printed elements are shown as photos in Figure 3, Figure 4 and Figure 5 and in Figure S2E with specific functions indicated in the respective captions. The elements include supports for motors, e.g., that can be easily attached to the syringe pump head valve, a lifting stand for a syringe pump (of interest if the pump is turned upside-down), motor attachments, stir-drivers, motor heads, flow adaptors to increase the functionality of the head-valve, and detection related elements. By this, we aim for an overview of how to use 3D printing for fit-for-purpose imperative as well as useful system components. Moreover, in addition to Figure 1 and Figure 2, they show a more visual impression of possible setups of Lab-In-Syringe systems. 

## 7. Conclusions

Lab-In-Syringe can be considered as a hybrid of flow-batch analysis and SIA. It diverts the typical setup of an SIA system from its intended use and breaks the unwritten rules of its parental technique. On the other hand, it is indisputable that LIS is a versatile alternative to tubing-based FT, that it allows automation of many sample pretreatment techniques, and shows specific advantages, most importantly, the ease to treat milliliter sample volumes. In this article, we have aimed for bringing LIS closer to newcomers and users of other FT by showing the different ways how this flexible technique can be used with high benefit. We hope that with time the LIS technique will spread even more and become, as SIA, recognized not only as “Tools of Inventive Science” but as standard laboratory automation technique. 

The similarity in sample volumes and required instrumentation (a syringe pump and a solution selector) between Lab-In-Syringe and autosampler systems allows the straightforward merging of both techniques, i.e., using autosampler instrumentation for LIS automation. 

In this work, we have shown the different modes of operation and examples with simple but useful additions and devices for LIS analyzer systems enabled by 3D printing. We believe that the LIS technique will undergo further diversification and that new developments will follow that also include this prototyping technique. 

## Supplementary Materials

The following items are available online, Figure S1: Details to LIS system adaptation; Figure S2: Preparation of a brush-less motor for in-syringe stirring; Figure S3: Typical signal dependencies in the optimization of DLLME protocols automated by Lab-In-Syringe.

## Abbreviations

DLLME	Dispersive Liquid-Liquid Microextraction
CPE	Cloud Point Extraction
FDM	Fused deposition modeling
FT	Flow techniques
LIS	Lab-In-Syringe
LPME	Liquid-phase microextraction
NdFeB	Neodymium alloy as used for strong magnets
SIA	Sequential Injection Analysis
STA	Stereolithography

## References

[1] Růžička J., Marshall G. (1990). Sequential injection: A new concept for chemical sensors, process analysis and laboratory assays. Anal. Chim. Acta.

[2] Economou A. (2005). Sequential-injection analysis (SIA): A useful tool for on-line sample handling and pre-treatment. Trends Anal. Chem..

[3] Horstkotte B., Miro M., Solich P. (2018). Where are modern flow techniques heading to?. Anal. Bioanal. Chem..

[4] Zagatto E.A.G., Rocha F.R.P. (2020). The multiple facets of flow analysis. A tutorial. Anal. Chim. Acta.

[5] Trojanowicz M., Kołacińska K. (2016). Recent advances in flow injection analysis. Analyst.

[6] Kolev S.D., Mc Kelvie I. (2008). Advances in Flow Injection Analysis and Related Techniques.

[7] Trojanowicz M. (2000). Flow injection analysis. Instrumentation and Applications.

[8] Hansen E.H., Růžička J. Flow Injection Analysis. Tutorial & News on Flow-Based Micronalytical Techniques. http://www.flowinjectiontutorial.com.

[9] Dias Diniz P.H.G., de Almeida L.F., Harding D.P., de Araújo M.C.U. (2012). Flow-batch analysis. Anal. Bioanal. Chem..

[10] Zagatto E.A.G., Carneiro J.M.T., Vicente S., Fortes P.R., Santos J.L.M., Lima J.L.F.C. (2009). Mixing chambers in flow analysis: A review. J. Anal. Chem..

[11] Prabhu G.R.D., Urban P.L. (2017). The dawn of unmanned analytical laboratories. Trends Anal. Chem..

[12] Maya F., Estela J.M., Cerdà V. (2012). Completely automated in-syringe dispersive liquid-liquid microextraction using solvents lighter than water. Anal. Bioanal. Chem..

[13] Maya F., Horstkotte B., Estela J.M., Cerdà V. (2012). Lab in a syringe: Fully automated dispersive liquid-liquid microextraction with integrated spectrophotometric detection. Anal. Bioanal. Chem..

[14] Horstkotte B., Alexovič M., Maya F., Duarte C.M., Andruch V., Cerdà V. (2012). Automatic determination of copper by in-syringe dispersive liquid–liquid microextraction of its bathocuproine-complex using long path-length spectrophotometric detection. Talanta.

[15] Suárez R., Horstkotte B., Duarte C.M., Cerdà V. (2012). Fully-automated fluorimetric determination of aluminum in seawater by in-syringe dispersive liquid-liquid microextraction using lumogallion. Anal. Chem..

[16] Horstkotte B., Suárez R., Solich P., Cerdà V. (2013). In-syringe stirring: A novel approach for magnetic stirring-assisted dispersive liquid-liquid microextraction. Anal. Chim. Acta.

[17] Henriquez C., Horstkotte B., Solich P., Cerdà V. (2013). In-syringe magnetic-stirring-assisted liquid-liquid microextraction for the spectrophotometric determination of Cr(VI) in waters. Anal. Bioanal. Chem..

[18] Horstkotte B., Suárez R., Solich P., Cerdà V. (2014). In-syringe magnetic stirring assisted dispersive liquid-liquid micro-extraction with solvent washing for fully automated determination of cationic surfactants. Anal. Meth..

[19] Šrámková I., Horstkotte B., Sklenářová H., Solich P., Kolev S.D. (2016). A novel approach to Lab-In-Syringe Head-Space Single-Drop Microextraction and on-drop sensing of ammonia. Anal. Chim. Acta.

[20] Horstkotte B., Lopez de los Mozos Atochero N., Solich P. (2018). Lab-In-Syringe automation of stirring-assisted room-temperature headspace extraction coupled online to gas chromatography with flame ionization detection for determination of benzene, toluene, ethylbenzene, and xylenes in surface waters. J. Chromatogr. A.

[21] Fikarová K., Horstkotte B., Sklenářová H., Švec F., Solich P. (2019). Automated continuous-flow in-syringe dispersive liquid-liquid microextraction of mono-nitrophenols from large sample volumes using a novel approach to multivariate spectral analysis. Talanta.

[22] Giakisikli G., Anthemidis A.N. (2018). Automatic pressure-assisted dual-headspace gas-liquid microextraction. Lab-in-syringe platform for membraneless gas separation of ammonia coupled with fluorimetric sequential injection analysis. Anal. Chim. Acta.

[23] Šrámková I.B., Horstkotte B., Solich P., Sklenářová H. (2014). Automated in-syringe single-drop head-space micro-extraction applied to the determination of ethanol in wine samples. Anal. Chim. Acta.

[24] Mitani C., Kotzamanidou A., Anthemidis A.N. (2014). Automated headspace single-drop microextraction via a lab-in-syringe platform for mercury electrothermal atomic absorption spectrometric determination after in situ vapor generation. J. Anal. Atom. Spectr..

[25] Dias A.C.B., Borges E.P., Zagatto E.A.G., Worsfold P.J. (2006). A critical examination of the components of the Schlieren effect in flow analysis. Talanta.

[26] Zhu X., Deng Y., Li P., Yuan D., Ma J. (2019). Automated syringe-pump-based flow-batch analysis for spectrophotometric determination of trace hexavalent chromium in water samples. Microchem. J..

[27] Ma J., Li P., Chen Z., Lin K., Chen N., Jiang Y., Chen J., Huang B., Yuan D. (2018). Development of an Integrated Syringe-Pump-Based Environmental-Water Analyzer ( iSEA) and Application of It for Fully Automated Real-Time Determination of Ammonium in Fresh Water. Anal. Chem..

[28] Paluch J., Kozak J., Wieczorek M., Kozak M., Kochana J., Widurek K., Konieczna M., Koscielniak P. (2017). Novel approach to two-component speciation analysis. Spectrophotometric flow-based determinations of Fe(II)/Fe(III) and Cr(III)/Cr(VI). Talanta.

[29] Wieczorek M., Rengevicova S., Świt P., Woźniakiewicz A., Kozak J., Kościelniak P. (2017). New approach to H-point standard addition method for detection and elimination of unspecific interferences in samples with unknown matrix. Talanta.

[30] Soares S., Melchert W.R., Rocha F.R.P. (2017). A flow-based procedure exploiting the lab-in-syringe approach for the determination of ester content in biodiesel and diesel/biodiesel blends. Talanta.

[31] Alexovič M., Horstkotte B., Solich P., Sabo J. (2017). Automation of dispersive liquid-liquid microextraction and related techniques. Approaches based on flow, batch, flow-batch and in syringe modes. Trends Anal. Chem..

[32] Alexovič M., Horstkotte B., Solich P., Sabo J. (2016). Automation of static and dynamic non-dispersive liquid phase microextraction. Part 1: Approaches based on extractant drop-, plug-, film- and microflow-formation. Anal. Chim. Acta.

[33] Alexovič M., Horstkotte B., Solich P., Sabo J. (2016). Automation of static and dynamic non-dispersive liquid phase microextraction. Part 2: Approaches based on impregnated membranes and porous supports. Anal. Chim. Acta.

[34] Clavijo S., del Rosario Brunetto M., Cerdà V. (2014). In-syringe-assisted dispersive liquid-liquid microextraction coupled to gas chromatography with mass spectrometry for the determination of six phthalates in water samples. J. Sep. Sci..

[35] Clavijo S., Avivar J., Suarez R., Cerdà V. (2016). In-syringe magnetic stirring-assisted dispersive liquid-liquid microextraction and silylation prior gas chromatography-mass spectrometry for ultraviolet filters determination in environmental water samples. J. Chromatogr. A.

[36] Villar M., Avivar J., Ferrer L., Borràs A., Vega F., Cerdà V. (2015). Automatic in-syringe dispersive liquid–liquid microextraction of 99Tc from biological samples and hospital residues prior to liquid scintillation counting. Anal. Bioanal. Chem..

[37] Vargas Medina D.A., Santos-Neto Á.J., Cerdà V., Maya F. (2018). Automated dispersive liquid-liquid microextraction based on the solidification of the organic phase. Talanta.

[38] Wang X., Xu G., Chen P., Sun Y., Yao X., Lv Y., Guo W., Wang G. (2018). Fully-automated magnetic stirring-assisted lab-in-syringe dispersive liquid–liquid microextraction for the determination of arsenic species in rice samples. RSC Advances.

[39] Sanchez R., Horstkotte B., Fikarová K., Sklenářová H., Maestre S., Miro M., Todoli J.L. (2017). Fully Automatic In-Syringe Magnetic Stirring-Assisted Dispersive Liquid Liquid Microextraction Hyphenated to High-Temperature Torch Integrated Sample Introduction System-Inductively Coupled Plasma Spectrometer with Direct Injection of the Organic Phase. Anal. Chem..

[40] González A., Avivar J., Cerdà V. (2015). Determination of priority phenolic pollutants exploiting an in-syringe dispersive liquid–liquid microextraction–multisyringe chromatography system. Anal. Bioanal. Chem..

[41] Horstkotte B., Fikarová K., Cocovi-Solberg D.J., Sklenářová39 H., Solich P., Miro M. (2017). Online coupling of fully automatic in-syringe dispersive liquid-liquid microextraction with oxidative back-extraction to inductively coupled plasma spectrometry for sample clean-up in elemental analysis: A proof of concept. Talanta.

[42] Suárez R., Clavijo S., Avivar J., Cerdà V. (2016). On-line in-syringe magnetic stirring assisted dispersive liquid-liquid microextraction HPLC--UV method for UV filters determination using 1-hexyl-3-methylimidazolium hexafluorophosphate as extractant. Talanta.

[43] Shishov A., Terno P., Moskvin L., Bulatov A. (2020). In-syringe dispersive liquid-liquid microextraction using deep eutectic solvent as disperser: Determination of chromium (VI) in beverages. Talanta.

[44] Timofeeva I., Shishov A., Kanashina D., Dzema D., Bulatov A. (2017). On-line in-syringe sugaring-out liquid-liquid extraction coupled with HPLC-MS/MS for the determination of pesticides in fruit and berry juices. Talanta.

[45] Pochivalov A., Vakh C., Garmonov S., Moskvin L., Bulatov A. (2020). An automated in-syringe switchable hydrophilicity solvent-based microextraction. Talanta.

[46] Frizzarin R.M., Portugal L.A., Estela J.M., Rocha F.R.P., Cerdà V. (2016). On-line lab-in-syringe cloud point extraction for the spectrophotometric determination of antimony. Talanta.

[47] Davletbaeva P., Falkova M., Safonova E., Moskvin L., Bulatov A. (2016). Flow method based on cloud point extraction for fluorometric determination of epinephrine in human urine. Anal. Chim. Acta.

[48] Maya F., Cabello C.P., Estela J.M., Cerdà V., Palomino G.T. (2015). Automatic In-Syringe Dispersive Microsolid Phase Extraction Using Magnetic-Metal Organic Frameworks. Anal. Chem..

[49] Gonzalez A., Avivar J., Maya F., Palomino Cabello C., Turnes Palomino G., Cerdà V. (2017). In-syringe dispersive mu-SPE of estrogens using magnetic carbon microparticles obtained from zeolitic imidazolate frameworks. Anal. Bioanal. Chem..

[50] Giakisikli G., Anthemidis A.N. (2017). An automatic stirring-assisted liquid-liquid microextraction system based on lab-in-syringe platform for on-line atomic spectrometric determination of trace metals. Talanta.

[51] Šrámková I.H., Horstkotte B., Fikarová K., Sklenářová H., Solich P. (2018). Direct-immersion single-drop microextraction and in-drop stirring microextraction for the determination of nanomolar concentrations of lead using automated Lab-In-Syringe technique. Talanta.

[52] Sasaki M.K., Souza P.A.F., Kamogawa M.Y., Reis B.F., Rocha F.R.P. (2019). A new strategy for membraneless gas-liquid separation in flow analysis: Determination of dissolved inorganic carbon in natural waters. Microchem. J..

