# Supplementary material for: The Automation Technique Lab-In-Syringe: A Practical Guide
Source: Molecules. 2020 Apr 1;25(7):1612. doi: 10.3390/molecules25071612 (PMC7181287; doi:10.3390/molecules25071612)
Supplement: Supplementary file 1 [file molecules-25-01612-s001.pdf]

Automation technique Lab-In-Syringe: A practical guide

Supplementary Materials

Burkhard Horstkotte, Petr Solich

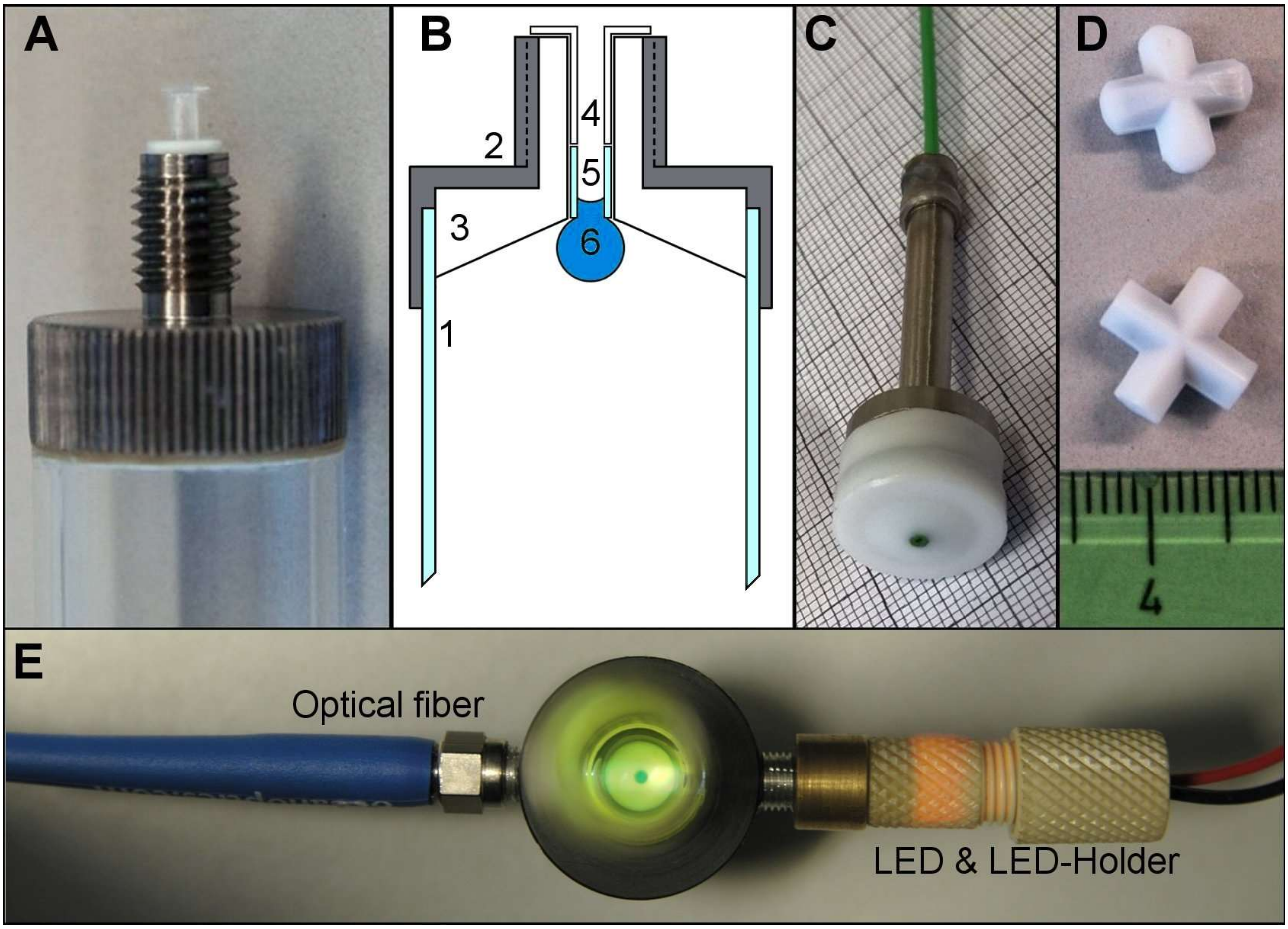

Figure S1. Details to LIS system adaptation. (**A**) Insert of a piece of PTFE tubing into syringe inlet to decrease diameter, (**B**) Decrease of inlet diameter as in A plus insert of glass capillary to increase inlet wettability and allow stable formation of a single drop for headspace extraction (1-Glass barrel, 2-Metallic casing, 3-crimp connection of PTFE, 4-PTFE tubing insert, 5-Glass capillary, 6-Drop) [19,23], (**C**) Syringe piston featured with secondary inlet flow line to syringe void, (**D**) Stirring cross, original and shaped to remain in the syringe inlet when using upside-down orientation enabling e.g. in-solvent-drop stirring [51]. (**E**) Lightpath using a fiber adaptor for in-syringe spectrophotometric detection visualized with fluorescein.

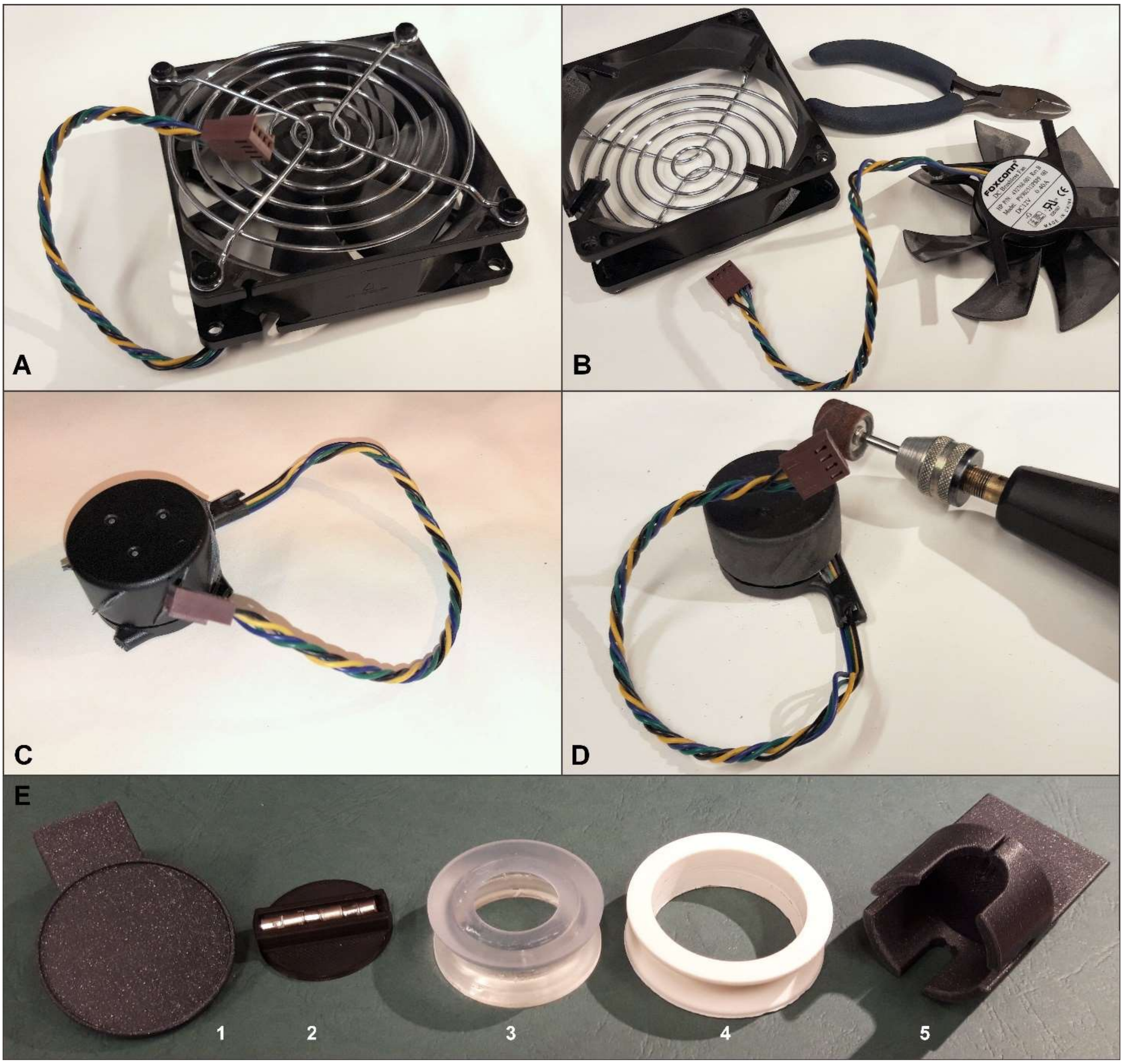

Figure S2. Preparation of a brush-less motor for in-syringe stirring. (**A**) Use of a computer van with pulse-width modulation control of velocity, (**B**) cutting away the supporting frame, (**C**) breaking off all wings, (**D**) sanding down excess ends to obtain a smooth surface shell. (**E**) 3D printed motor attachments for NdFeB magnet placement (**E2**) or pulley-wheel function (**E3, E4**) and motor supports as shown in Figures 3 and 4 (**E1, E5**).

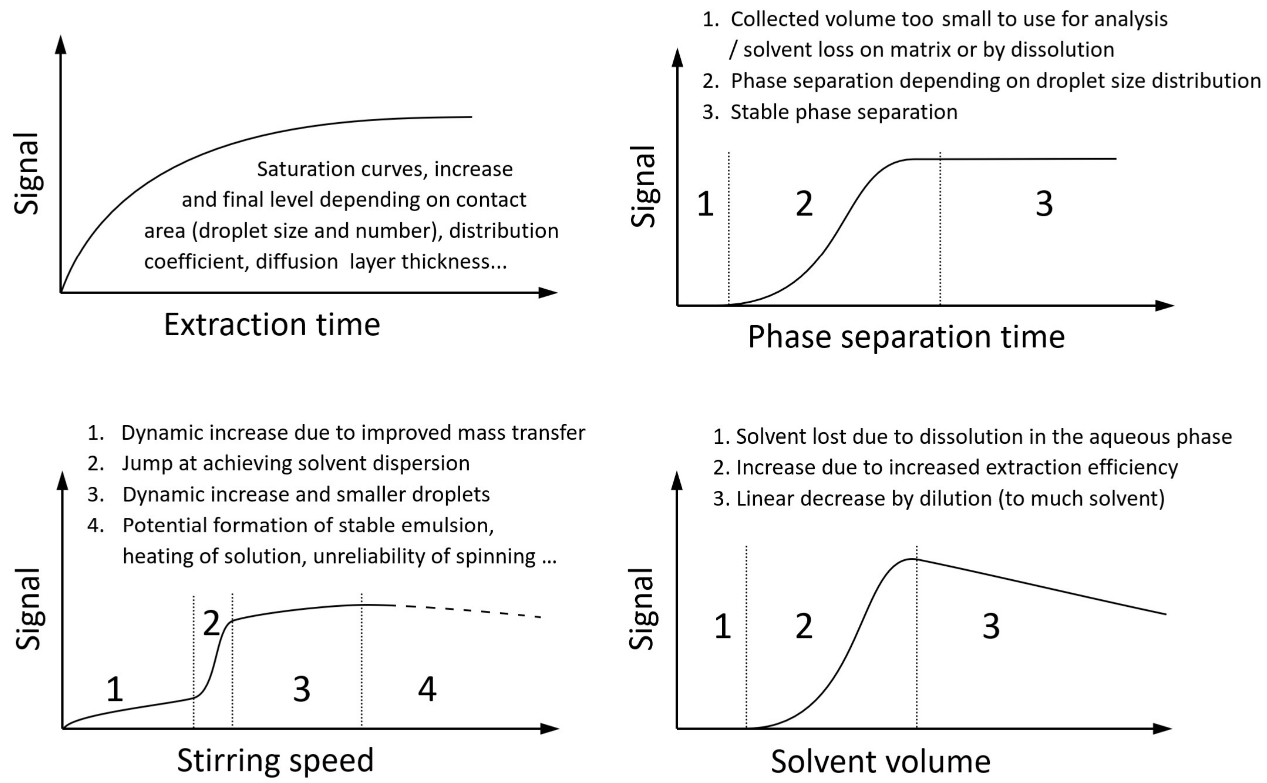

Figure S3. Typical signal dependencies in the optimization of DLLME protocols automated by Lab-In-Syringe.
